# Sleep duration and cardiovascular disease risk in type 2 diabetes: A prospective cohort study

**DOI:** 10.1371/journal.pone.0337232

**Published:** 2025-11-18

**Authors:** Zhi Jin, Haiyan Wang, Hai Bin Wen

**Affiliations:** 1 Department of Neurology, Shanghai Fifth People’s Hospital, Fudan University, Shanghai, China; 2 Center of Community-Based Health Research, Fudan University, Shanghai, China; 3 Department of Endocrinology, The Eighth People’s Hospital of Qingdao, Qingdao, China; 4 Department of Nephrology, Jiang bin Hospital of Guangxi Zhuang Autonomous Region, Nanning, China; National Healthcare Group, SINGAPORE

## Abstract

**Background:**

Cardiovascular disease (CVD) remains the leading cause of mortality in Type 2 Diabetes Mellitus (T2DM) patients. While sleep duration has emerged as a potential modifiable risk factor for CVD, evidence from prospective studies among T2DM patients remains scarce and inconsistent. We aimed to investigate the association between sleep duration and incident CVD risk among Chinese adults with T2DM.

**Methods:**

We conducted a prospective cohort study using data from the China Health and Retirement Longitudinal Study (CHARLS) (2015–2018). Sleep duration was assessed through validated questionnaires at baseline. Incident CVD was defined as the first occurrence of physician-diagnosed coronary heart disease, stroke, or heart failure during follow-up. Logistic regression models were employed to calculate odds ratios (ORs) and 95% confidence intervals (CIs) for the risk of CVD, with adjustments made for potential confounding variables.

**Results:**

Among 1,360 participants with T2DM, the mean sleep duration was 6.45 ± 1.95 hours. During follow-up, 237 (17.43%) participants reported new-onset CVD. In the fully adjusted model, a significant inverse association was found between sleep duration and CVD risk (OR: 0.92, 95% CI: 0.86–0.99, P = 0.03). Notably, individuals with long sleep duration (>9 hours) had a significantly decreased risk of CVD (OR: 0.36, 95% CI: 0.15–0.85, P = 0.02) compared to those with shorter sleep durations.

**Conclusion:**

Our findings indicate an inverse association between sleep duration and the risk of new-onset cardiovascular disease in Chinese adults with type 2 diabetes mellitus, with longer sleep duration associated with lower CVD risk.

## Introduction

Type 2 diabetes mellitus (T2DM) is a global health concern, with a rapidly increasing prevalence, particularly in developing countries such as China [1]. A nationwide survey conducted in China from 2015 to 2017 revealed that the prevalence of diabetes among Chinese adults had reached 12.8% [2]. Individuals with T2DM are at an elevated risk of cardiovascular disease (CVD), which remains the leading cause of morbidity and mortality in this population [3–5]. While traditional risk factors for CVD in T2DM, such as hyperglycemia, hypertension, and dyslipidemia, are well-established [4,6], emerging evidence suggests that sleep duration may play a crucial role in modulating cardiovascular risk [7–10].

Epidemiological studies have shown that sleep disorders, usually attributed to work stress and environmental deterioration, are on the rise worldwide [11,12]. Particularly in China, sleep patterns have undergone significant changes due to rapid urbanization. Furthermore, Type 2 diabetes mellitus significantly impacts sleep through multiple mechanisms, including nocturia, neuropathic pain, and glucose fluctuations, leading to shorter sleep duration and altered sleep architecture compared to non-diabetic individuals [13]. The biological mechanisms linking sleep duration to CVD in T2DM are multifactorial, involving sympathetic nervous system activation, systemic inflammation, endothelial dysfunction, and hormonal readiness [14,15]. These pathways may be amplified in T2DM patients due to pre-existing metabolic dysregulation, creating a vulnerable substrate for cardiovascular events. Existing data among people with diabetes have linked sleep duration and CVD risk both directly and indirectly, as short and long sleep durations were associated with poor glycemic control, worse cardiometabolic risk profiles, and a higher prevalence of CVD [16–18].

Sleep is a fundamental physiological process that influences numerous metabolic pathways and cardiovascular functions [19]. Alterations in sleep duration have been associated with various adverse health outcomes, including obesity, insulin resistance, and hypertension [20,21]. Recent studies have provided compelling evidence linking sleep duration to cardiovascular risk in individuals with T2DM. Research using data from the UK Biobank revealed a U-shaped association, where both short (≤5 hours) and long (≥10 hours) sleep durations were linked to higher risks of atherosclerotic cardiovascular disease and mortality, compared to the reference sleep duration of 7 hours [18]. Corroborating these findings, a study from Taiwan observed a J-shaped association between sleep duration and mortality risk in T2DM patients, with the lowest risk among those sleeping 5–7 hours per night [22].

Understanding the sleep-CVD relationship in Chinese T2DM patients is particularly important given population-specific characteristics. Chinese populations exhibit distinct KCNQ1 variations affecting diabetes susceptibility [23] and different sleep patterns [24] with marked occupational variations [25].These genetic and lifestyle differences warrant population-specific investigation rather than extrapolating from Western cohorts. Studies have varied considerably in their design characteristics, including cross-sectional versus longitudinal approaches, different age groups, and varying baseline health status of participants, which may explain the inconsistent associations reported. To address these gaps, we utilized the China Health and Retirement Longitudinal Study (CHARLS) [26] to examine sleep-CVD associations in mainland Chinese adults with T2DM. Our study complements existing systematic reviews by providing the prospective examination of this relationship in a mainland Chinese population with unique genetic background and lifestyle factors that may modify the sleep-CVD association observed in Western populations.

## Method

### Research design and methods

#### Study population.

This longitudinal investigation utilizes data from the China Health and Retirement Longitudinal Study (CHARLS), a nationally representative survey of Chinese residents aged 45 and above, conducted from 2008 to 2018. The CHARLS encompasses comprehensive assessments of demographic characteristics, health status and functionality, socioeconomic indicators, and retirement-related information. The study’s sampling methodology employs a multistage probability approach, with participants recruited from 28 Chinese provinces [26].

**Fig 1:**
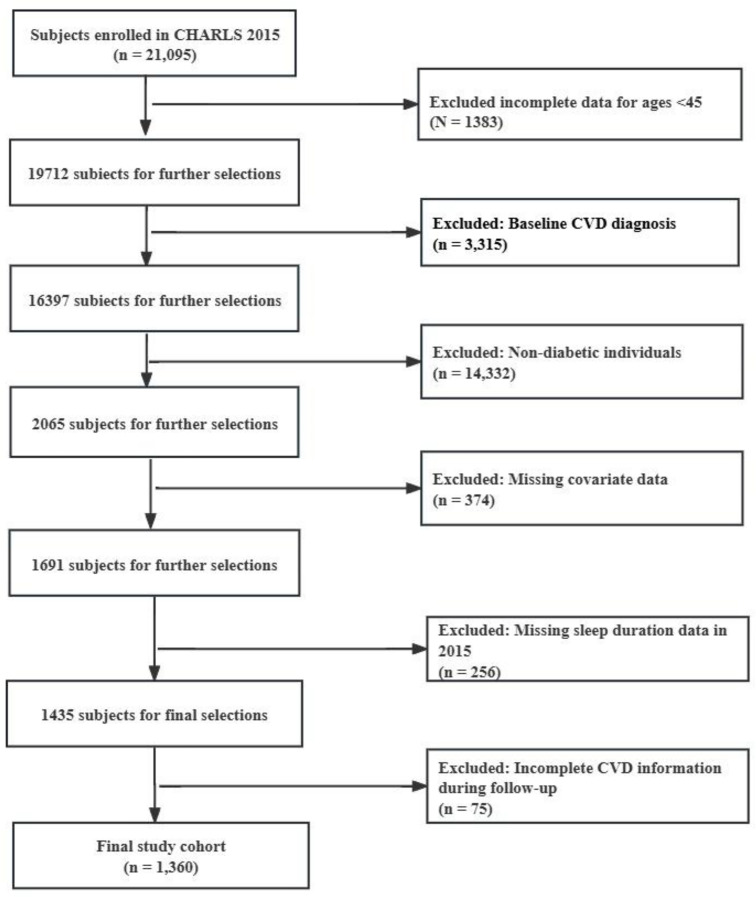
Flow Chart of Study Participant Selection.

The present analysis established 2015 as the baseline year, with follow-up outcomes assessed in 2018. We initially identified 21,095 participants with type 2 diabetes at baseline. Diabetes diagnosis was established through one or more of the following criteria: self-reported physician diagnosis, use of hypoglycemic medications, fasting blood glucose (FBG) ≥ 126 mg/dL, or glycated hemoglobin (HbA1c) level ≥ 6.5% [27]. To refine our study cohort, we applied several exclusion criteria: individuals aged below 45 years (n = 1,383), participants with a baseline diagnosis of cardiovascular disease (CVD) (n = 3,315), non-diabetic individuals (n = 14,332), cases with missing covariate data (n = 374), We employed complete case analysis (listwise deletion) for missing data, consistent with standard practice in similar epidemiological studies, as the overall missingness rate was low and data were missing at random. participants lacking sleep data in 2015 (n = 256), and individuals with incomplete CVD information during follow-up (n = 75). This rigorous selection process yielded a cohort specifically tailored to our research objectives, comprising middle-aged and older adults with type 2 diabetes and no pre-existing CVD at baseline. The full process of participants’ selection is depicted in Fig 1.

#### Ethics statement.

The China Health and Retirement Longitudinal Study (CHARLS) was conducted in strict adherence to ethical standards for human subject research. The study protocol underwent comprehensive ethical review and received approval from the Biomedical Ethics Review Committee of Peking University (IRB00001052–11015). This approval ensures compliance with international ethical guidelines for biomedical research involving human participants. All subjects enrolled in the cohort studies provided written informed consent prior to participation.

#### Ascertainment of outcomes.

The primary endpoint of this study was the incidence of cardiovascular disease (CVD), defined as a composite outcome of coronary heart disease and stroke. A validated methodology was used to identify new-onset CVD, based on standardized questionnaires administered by trained interviewers. These questionnaires were aligned with leading international aging surveys to ensure data comparability and reliability. Participants were classified as having experienced a new-onset CVD event if they answered affirmatively to either of the following physician-diagnosed conditions during the follow-up period: “Have you been diagnosed with a heart attack, coronary heart disease, angina, congestive heart failure, or other heart problems?” or “Have you been diagnosed with a stroke?” [28,29].

#### Assessment of exposure.

Sleep duration, the primary exposure variable in this study, was assessed at baseline through self-reported measures obtained during face-to-face interviews. Participants were queried about their nocturnal sleep patterns using a standardized, open-ended question: “During the past month, how many hours of actual sleep did you get at night (average hours for one night)?” The participants were divided into 3 groups according to sleep duration (<=6,6–9, > 9hours per night) for the analyses [30].

#### Covariates.

At baseline, comprehensive data on socio-demographic and health-related factors were collected using a structured questionnaire. Socio-demographic variables included age, sex, education level (elementary school or below, secondary school, college or above), marital status (cohabiting or living alone), and residence (rural or urban). Health-related factors included smoking and drinking status (never, current, or former), hypertension, and dyslipidemia. Hypertension was defined as systolic blood pressure ≥140 mmHg, diastolic blood pressure≥90 mmHg, self-reported diagnosis, or current use of antihypertensive medications [31]. Chronic kidney disease (CKD) was determined by self-reported physician diagnosis or an estimated glomerular filtration rate of < 60 ml/min/1.73 m² [32].Dyslipidemia was defined as a self-reported diagnosis or by meeting any of the following criteria: total cholesterol≥6.2 mmol/L, LDL-C ≥ 4.1 mmol/L, triglycerides≥2.3 mmol/L, or HDL-C < 1.0 mmol/L [33]. Blood samples were collected after overnight fasting by trained medical staff, stored at −20°C, and transported to Beijing for analysis. Glycosylated hemoglobin (HbA1c) levels were measured using boronate affinity high-performance liquid chromatography.

#### Statistical analysis.

Data are presented as mean ± standard deviation (SD) for continuous variables and as numbers with percentages for categorical variables. Differences among sleep duration groups were assessed using χ2 tests for categorical variables and one-way ANOVA for normally distributed continuous variables.

To examine the association between sleep duration and CVD risk, we employed logistic regression analysis, systematically adjusting for potential confounders across distinct models. Three models were constructed: Model 1 (unadjusted); Model 2 (adjusted for sex, age, and BMI); and Model 3 (adjusted for sex, age, BMI, marital status, education, residence, smoking status, drinking status, HbA1c, hypertension, dyslipidemia, and chronic kidney disease). All covariates were coded as displayed in Table 1, with age and HbA1c as continuous variables and others as categorical. The first category in Table 1 served as the reference group in regression analyses. Effect sizes with 95% confidence intervals were reported for each model.

**Table 1:** Baseline Characteristics of Study Participants Stratified by Sleep Duration Categories.

	Q1 (<=6)	Q2 (6–9)	Q3 (>9)	P-value
**Age(years)**	62.40 ± 8.27	61.38 ± 8.76	63.11 ± 10.66	0.06
**Sex n (%)**				0.22
female	382(56.93)	330(53.48)	45(62.50)	
male	289(43.07)	287(46.52)	27(37.50)	
**BMI**				**<0.01**
Normal (<=24)	291(43.37)	224(36.30)	29(40.28)	
Overweight (>24,<=28)	273(40.69)	253(41.00)	24(33.33)	
Obese (>28)	107(15.95)	140(22.69)	19(26.39)	
**Marital status n (%)**				0.61
Cohabitation	555(82.71)	523(84.76)	60(83.33)	
Solitude	116(17.29)	94(15.24)	12(16.67)	
**Education n (%)**				0.14
Elementary school and below	463(69.00)	414(67.10)	58(80.56)	
high school	196(29.21)	187(30.31)	14(19.44)	
college and higher	12(1.79)	16(2.59)		
**Rresidence n (%)**				**0.01**
rural	412(61.40)	351(56.89)	53(73.61)	
urban	259(38.60)	266(43.11)	19(26.39)	
**Smoking status n (%)**				0.96
never	404(60.21)	371(60.13)	46(63.89)	
current	173(25.78)	155(25.12)	16(22.22)	
former	94(14.01)	91(14.75)	10(13.89)	
**Drinking status n (%)**				0.14
never	461(68.70)	402(65.15)	54(75.00)	
current	165(24.59)	158(25.61)	11(15.28)	
former	45 (6.71)	57 (9.24)	7 (9.72)	
**HBA1C (%)**	7.18 ± 1.73	7.32 ± 1.71	7.28 ± 1.79	0.31
**Hypertension n (%)**				0.47
No	190(47.62)	362(44.04)	65(46.76)	
Yes	209(52.38)	460(55.96)	74(53.24)	
**Dyslipidemia n (%)**				0.75
No	459(68.41)	410(66.45)	49(68.06)	
Yes	212(31.59)	207(33.55)	23(31.94)	
**Chronic kidney disease n (%)**				0.25
No	615(91.65)	580(94.00)	66(91.67)	
Yes	56(8.35)	37(6.00)	6(8.33)	

Table Results Format: (N) Mean (SD)/ N (%).

Potential non-linear relationships were investigated using generalized additive models (GAM) with thin plate regression splines (mgcv package). Smoothing parameters were automatically selected using generalized cross-validation (GCV). Non-linearity was assessed by visual inspection of smoothed curves and effective degrees of freedom (EDF). When curves showed inflection points, two-piecewise logistic regression models were fitted with optimal inflection points identified by recursive algorithm, and log-likelihood ratio tests confirmed improved fit. Subgroup analyses were conducted using stratified binary logistic regression. Continuous variables were categorized based on clinical cut-points or tertiles for interaction testing. Effect modification was assessed using likelihood ratio tests.

All analyses were performed using R statistical software (version 4.2.0, R Foundation for Statistical Computing, Vienna, Austria), FreeStatistics software version 1.8 and EmpowerStats (X&Y Solutions, Inc., Boston, MA, USA). Two-sided P values < 0.05 were considered statistically significant.

## Result

Table 1 presents the baseline characteristics of the study population stratified by sleep duration. Significant differences were observed in BMI distribution (p < 0.01) and place of residence (p = 0.01) among sleep duration groups. The > 9 hours sleep group had a higher proportion of obese individuals (26.39%) and rural residents (73.61%) compared to other groups. No significant differences were found in age, sex, education, marital status, smoking, drinking, HbA1c levels, hypertension, dyslipidemia, or chronic kidney disease across sleep duration categories.

Table 2 presents the incidence of new cardiovascular disease (CVD) events across different sleep duration categories during the 2018 follow-up. Among participants sleeping≤6 hours per night (Q1), 18.78% (n = 126) developed CVD. In the 6–9 hours sleep group (Q2), the incidence was slightly lower at 17.02% (n = 105). Notably, the > 9 hours sleep group (Q3) showed the lowest incidence of CVD at 8.33% (n = 6). Although these differences approached statistical significance (p = 0.08), they did not reach the conventional threshold of p < 0.05.

**Table 2:** Incidence of New-Onset Cardiovascular Disease in 2018 Follow-up by Sleep Duration Groups.

	Q1(<=6)	Q2(6–9)	Q3(>9)	P-value
**Cardiovascular disease n (%)**				0.08
No	545(81.22)	512(82.98)	66(91.67)	
Yes	126(18.78)	105(17.02)	6 (8.33)	

Table 3 presents the association between sleep duration and cardiovascular disease risk in patients with type 2 diabetes across different adjustment models. Sleep duration as a continuous variable showed a consistent, significant inverse association with cardiovascular disease risk across all models (Fully adjusted OR: 0.92, 95% CI: 0.86–0.99, p = 0.03). When categorized, compared to sleep duration≤6 hours, sleeping >9 hours was associated with significantly lower cardiovascular disease risk in all models. This association remained robust after full adjustment (OR: 0.36, 95% CI: 0.15–0.85, p = 0.02).

**Table 3:** Association of Sleep Duration with Cardiovascular Disease Risk in Type 2 Diabetes Patients: Analysis Using Different Models.

	Non-adjusted	Adjust I	Adjust II
Sleep duration	0.92 (0.86, 0.99) 0.03	0.920(0.86, 0.99) 0.02	0.92(0.86, 0.99) 0.03
Sleep duration subgroups			
<=6	1.0(Ref)	1.0(Ref)	1.0(Ref)
>6, <=9	0.89 (0.67, 1.18) 0.41	0.88 (0.66, 1.17) 0.38	0.88 (0.65, 1.18) 0.39
>9	0.39 (0.17, 0.93) 0.03	0.37 (0.16, 0.87) 0.02	0.36 (0.15, 0.85) 0.02

Note:

Data are presented as odds ratio (95% confidence interval).

Non-adjusted model: No covariates adjusted.

Adjust I model: Adjusted for sex, age, and BMI.

Adjust II model: Adjusted for sex, age, BMI, marital status, education, residence, smoking status, drinking status, HbA1c, hypertension, dyslipidemia and chronic kidney disease.

**Fig 2:**
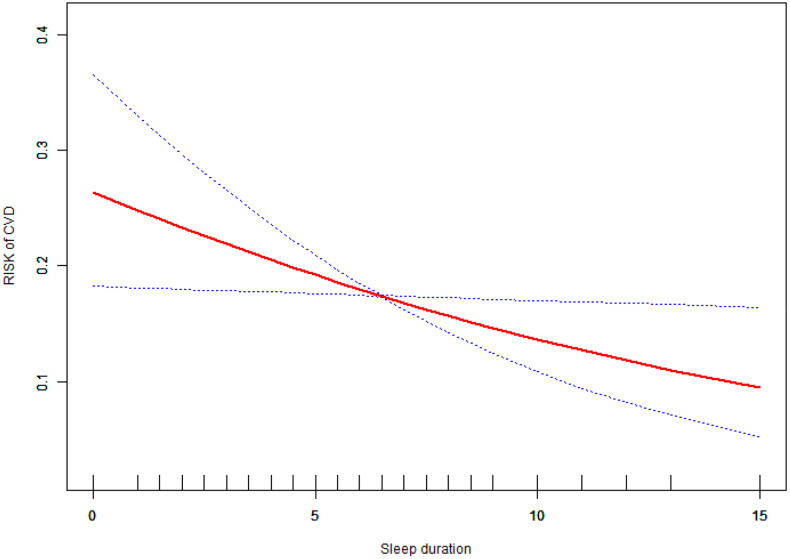
Linear relationship between sleep duration and cardiovascular disease (CVD) risk in type 2 diabetes patients.

As illustrated in Fig 2, the smooth curve plot demonstrates a clear linear relationship between sleep duration and the risk of cardiovascular disease (CVD) in patients with type 2 diabetes. As sleep duration increases, there is a consistent decrease in CVD risk. The red line shows a steady, downward slope from left to right, indicating that longer sleep duration is associated with lower CVD risk. This linear trend is maintained across the entire range of sleep durations shown (0–15 hours).The blue dotted lines represent the 95% confidence intervals, which remain relatively narrow throughout most of the sleep duration range, suggesting good precision in the estimate. However, the confidence intervals widen slightly at the extremes of sleep duration, particularly beyond 12 hours, indicating increased uncertainty in these areas. This linear relationship suggests that each additional hour of sleep is associated with a consistent reduction in CVD risk for patients with type 2 diabetes, within the observed range of sleep durations.

**Fig 3:**
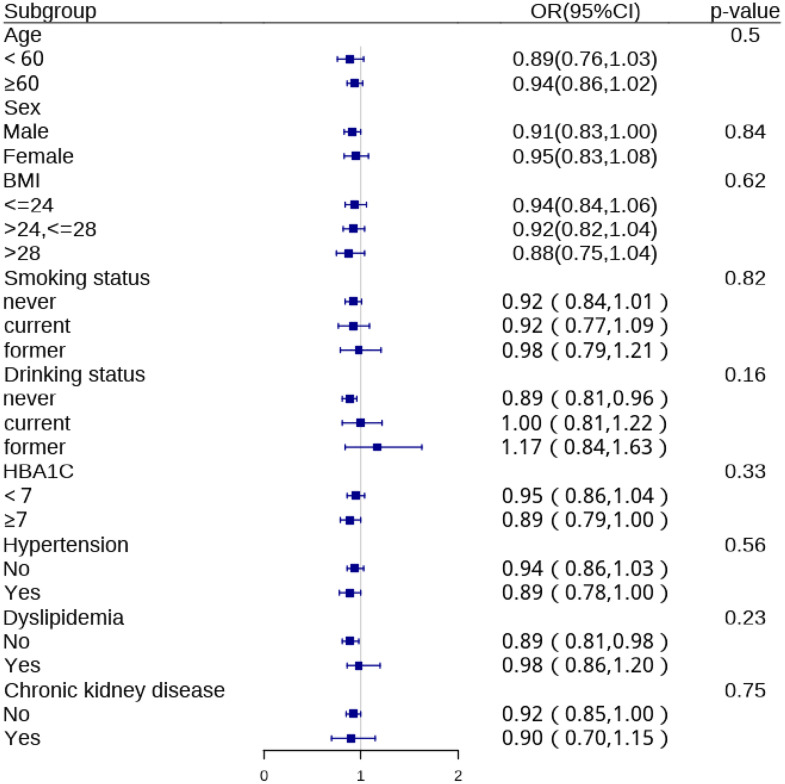
Subgroup Analysis of Sleep Duration and Cardiovascular Disease Risk in Type 2 Diabetes Patients. Figure note: forest plot showing odds ratios (ORs) and 95% confidence intervals (CIs) for the association between sleep duration (per 1-hour increase) and cardiovascular disease risk across various subgroups. ORs were calculated using logistic regression models, adjusting for all covariates except the stratification variable.

Fig 3 presents the subgroup analyses of the association between sleep duration and cardiovascular disease risk. The inverse association was consistent across most examined subgroups, including age, sex, BMI, smoking status, drinking status, HbA1c levels, hypertension, dyslipidemia, and chronic kidney disease status. No statistically significant interactions were observed for any subgroups (all p for interaction > 0.05), suggesting that the association between longer sleep duration and cardiovascular disease risk in patients with type 2 diabetes is relatively consistent across various patient characteristics.

## Discussion

This study, utilizing data from the China Health and Retirement Longitudinal Study (CHARLS), reveals that increased sleep duration is associated with a reduced risk of CVD events in T2DM patients (OR: 0.92, 95% CI: 0.86–0.99, P = 0.03). This association persisted after adjusting for potential confounding factors. Notably, longer sleep duration (>9 hours) was associated with lower CVD risk. However, given the observational design and self-reported measures, these findings reflect associations rather than causal effects, and cannot establish that longer sleep duration confers cardiovascular protection. This finding contrasts with previous studies reporting U-shaped or J-shaped associations between sleep duration and CVD risk [18,34–37]. First, our study focused on middle-aged and older Chinese adults with T2DM, whereas prior studies predominantly included Western or general populations. Diabetes-related metabolic dysregulation may modify the sleep-CVD relationship [38,39]. Second, methodological differences including sleep measurement instruments, categorization thresholds, and covariate adjustment strategies may contribute to heterogeneous findings. Third, residual confounding from unmeasured factors such as sleep quality, sleep disorders, or depression cannot be excluded.

While Table 2 presents descriptive CVD incidence rates across sleep duration categories using chi-square analysis, which shows a trend (p = 0.08) but does not reach statistical significance, our main findings are based on logistic regression analysis (Table 3) and smooth curve fitting (Fig 2). The logistic regression provides greater statistical power by treating sleep duration as a continuous variable and adjusting for confounding factors, revealing the significant linear inverse association (OR: 0.92, 95% CI: 0.86–0.99, P = 0.03). Our findings contribute to existing knowledge by demonstrating that the sleep-CVD relationship in Chinese T2DM patients follows a linear protective pattern rather than the U-shaped associations commonly reported in Western populations, suggesting important population-specific differences that challenge universal applicability of current sleep recommendations. Regarding clinical implications, while our data suggest potential cardiovascular benefits of longer sleep duration, recommendations should be made cautiously, considering that longer sleep should represent natural, restorative sleep rather than excessive sleep secondary to underlying illness or sleep disorders. Future research should focus on prospective studies with objective sleep measurements, mechanistic investigations of population-specific differences, and intervention trials to establish causality.

Several physiological mechanisms may explain the observed association. First, adequate sleep contributes to improved glycemic control by modulating insulin sensitivity and glucose metabolism [30,40], and optimal glycemic control reduces cardiovascular risk in diabetic patients [41]. Second, sufficient sleep attenuates systemic inflammation, with evidence showing sleep restriction increases pro-inflammatory markers (IL-6, CRP) implicated in CVD [42,43]. However, whether these mechanisms fully explain the protective association in T2DM populations, and whether longer sleep durations confer additional benefits, requires further investigation.

Our study benefits from several strengths, including the use of a large, nationally representative sample and a longitudinal design, which allows for the assessment of temporal relationships between sleep duration and CVD events. The adjustment for multiple potential confounders enhances the validity of our findings. Several limitations warrant acknowledgment. First, both sleep duration and CVD outcomes were self-reported, potentially introducing recall bias and misclassification. Second, we lacked data on sleep quality and sleep disorders (particularly sleep apnea), which are important confounders in T2DM populations. Third, the three-year follow-up may not fully capture long-term cardiovascular risk. Fourth, reverse causality cannot be excluded, as subclinical CVD at baseline could have influenced sleep patterns. Finally, residual confounding and the observational design preclude causal inferences. Future studies with objective sleep measures, medical record-verified outcomes, and longer follow-up are needed. The clinical implications of our findings are significant. Sleep duration may represent an important modifiable risk factor for CVD in T2DM patients. Healthcare providers should consider incorporating sleep assessment and management into the routine care of individuals with T2DM. Encouraging adequate sleep duration, potentially even longer than the general recommendation of 7–9 hours, may be beneficial for this high-risk population.

## Conclusion

In conclusion, our study identifies an inverse association between longer sleep duration and cardiovascular disease risk in Chinese adults with type 2 diabetes mellitus. However, the observational design with self-reported measures precludes causal inference. These findings suggest sleep duration as a potential area for further investigation. Validation through prospective studies with objective sleep measures and verified cardiovascular outcomes, as well as randomized controlled trials, is needed to establish causality and elucidate mechanisms before recommending changes to clinical practice or public health guidelines.

### Consent for publication

All authors have reviewed and approved the manuscript for publication.
